# The Kids Are Alright (?). Infants’ Development and COVID-19 Pandemic: A Cross-Sectional Study

**DOI:** 10.3389/ijph.2022.1604804

**Published:** 2022-06-20

**Authors:** Eleonora Ferrari, Lucia Palandri, Laura Lucaccioni, Giovanna Talucci, Erica Passini, Viola Trevisani, Elena Righi

**Affiliations:** ^1^ Department of Biomedical, Metabolic and Neural Sciences, University of Modena and Reggio Emilia, Modena, Italy; ^2^ Pediatric Unit, Department of Medical and Surgical Sciences for Mothers, Children and Adults, University of Modena and Reggio Emilia, Modena, Italy; ^3^ Department of Medical and Surgical Sciences for Mothers, Children and Adults, Postgraduate School of Pediatrics, University of Modena and Reggio Emili, Modena, Italy; ^4^ Neonatal Intensive Care Unit, Department of Medical and Surgical Sciences for Mothers, Children and Adults, University of Modena and Reggio Emilia, Modena, Italy

**Keywords:** public health, SARS-CoV-2, infant, Griffiths development scales, child development, mental processes, psychomotor performance, physical distancing

## Abstract

**Objectives:** The study aimed to assess and compare the global development in six-month-old infants before and during the pandemic restrictive social distancing measures.

**Methods:** This cross-sectional nested study involved infants assessed through the Griffiths Scales of Child Development (GSCD) between September 2019 and April 2021. Infants were classified in a pre-COVID or a COVID group, considering the evaluation date and the restrictive measures in place. GSCD subscales and General Development Scores (GDS) were calculated and compared.

**Results:** One hundred and four healthy term-born infants were evaluated. GDS in the COVID group (n:70; median: 94; IQR: 90–100) appeared significantly lower than in the pre-COVID group (n:34; median: 98; IQR: 97–103; *p* < 0.001). Language and personal-social-emotional subareas scores appeared the most affected. A decreasing trend of GDS along with the severity of restriction was observed.

**Conclusion:** A reduction in infant development scores was observed during pandemic social distancing. Further studies are needed to systematize these findings and to address effective public health policies for infants and families during long-term forced isolation periods.

## Introduction

On 11 March 2020, the Italian government imposed a national lockdown to reduce the spread of severe acute respiratory syndrome coronavirus 2 (SARS-CoV-2) [1]. After the pandemic outbreak, a succession of multiple waves took place, leading to the implementation of different restrictive measures in order to curtail the diffusion of the virus. All familial profiles are at risk when drastic changes—such as confinement—occur, especially those with higher susceptibility to socioeconomic factors and previous internal psychological problems [2].

While children’s health is clearly a key investment for public health [3], it is well established that unpredictable adverse events (such as war, natural disasters, and pandemics) have a huge impact on familial relationships and can affect the social and environmental background of a growing child [4]. Epidemics and pandemics, such as COVID-19, bring potential risks to child development in many fields and shape up to be possible “adverse childhood experiences” (ACEs) [5].

Many exogen stressors emerged during COVID-19 [6], threatening families’ financial security and routine, as well as preschoolers’ access to nurseries. Social restrictions caused children to spend time alone with their family nuclei, and newborns’ social and language abilities are known to be strongly influenced by their social environment. Evidence suggests that the amount of speech children hear, lexical diversity, and inclusion of language goals in play have a major role in children’s cognitive, language, and social development t [7].

Assessing the potential impact of social restrictions due to the pandemic on children’s development is an important public health goal. Although the association between isolation due to the COVID-19 pandemic and adverse mental health outcomes has been established in adult and adolescent cohorts [8–12], few studies [13, 14] have examined the association between changes in psychological functioning and home confinement in pre-school children.

The aim of the present study is to describe the global development as measured by the Griffiths Scales of Child Development (GSCD) [4, 15] of a cohort of six-month-old infants which were evaluated partially before and partially during the COVID-19 pandemic outbreak in periods characterized by different levels of restrictive measures.

## Methods

### Study Design and Participants

This is a cross-sectional analysis nested on an ongoing prospective cohort study. The main study started in 2019 with the aim of investigating the relationship between exposure to phthalates and anthropometric and neurocognitive development in Italian children during the first 3 years of life. Study subjects were enrolled at birth and follow-up visits were planned at 3, 6, 15, and 36 months. Neurocognitive development evaluation was programmed from the 6 months follow-up, onwards [16].

The study was approved by Area Vasta Emilia Nord Ethics Committee (2018/num715). Informed consent to participation and publication was obtained from caregivers of all individual participants included in the study.

Newborns with mothers of legal age (>18 years old) at delivery, who had an understanding of the Italian language and had a physiological single-child pregnancy were eligible for participation in the main cohort study. Other inclusion criteria were delivery occurred at term (37–41 weeks), giving birth to an appropriate-for-gestational-age (AGA) infant, with an Apgar score >7 at 5 minutes.

Invitation and enrollment took place in a second-level university hospital in Modena (Italy) between March 2019 and October 2020. Sample size calculation was performed for the main study, and it was estimated that about 200 newborns were needed. Therefore, we enrolled 197 mothers and child pairs. Among them, 104 infants completed the global development assessment at 6 months, and this determined the sample size for the present cross-sectional study. The six-month follow-up underwent from September 2019 to April 2021. The first local SARS-CoV-2 case in Italy was diagnosed on 21st February 2020. This unexpected situation gave us the opportunity to describe and compare global development in six-month-old healthy infants submitted to different levels of social restrictions.

### Variables

Regarding outcome, the global child development was assessed by the GSCD (third edition) [4, 15] a widely used [17–19] specialistic tool exploring five key evolutionary areas: the “Foundations of Learning” subscale (scale A), the “Language and Communication” subscale (scale B), the “Eye and Hand Coordination” subscale (scale C), the “Personal-Social-Emotional” subscale (scale D) and the “Gross Motor” subscale (scale E). Subscale scores and a total score, the “General Development score” (GDS) were calculated. Subscale and GDS raw scores were standardized for sex and age according to GSCD normative scoring tables and guidelines, and standardized “Developmental Quotient (DQ)” scores were produced. In the present study, we used the Italian validated version GSCD questionnaire addressed to 0–12 months children and the normative scoring tables produced for the Italian population [20]. The Griffiths III administration manual [15] lists seven classes for scoring interpretation: Extremely high (DQ ≥ 130), High (DQ = 120–129), Above average (DQ = 110–119), Average (DQ = 90–109), Below average (DQ 80–89), At the limits (DQ = 70–79), Extremely low (DQ ≤ 69). Accordingly, we classified the observed standardized DQ into three categories: Above average (DQ ≥ 110), Average (DQ = 90–109) and Below average (DQ ≤ 89) scores.

GSCD is a play-based test determining the level of development in infants aged 1 month to 5 years and 11 months (71 months) by observing their performance in a variety of standardized activities.

In the present study, a trained team of pediatricians and a psychologist administered the GSCD at the six-month follow-up visit as scheduled in the main longitudinal study. The mean duration of the visit was 1 h 15 and the mean time required for GSCD administration was 45 min. During assessment, the primary focus of specialists is on observing the child interacting and playing with the instruments provided in the GSCD administration kit. After February 2020 due to pandemic restrictions, some of the 6-month follow-up visits were performed online. Staff and mothers were trained to accurately reproduce the procedures and assessments of in-person GSCD visits.

**FIGURE 1:**
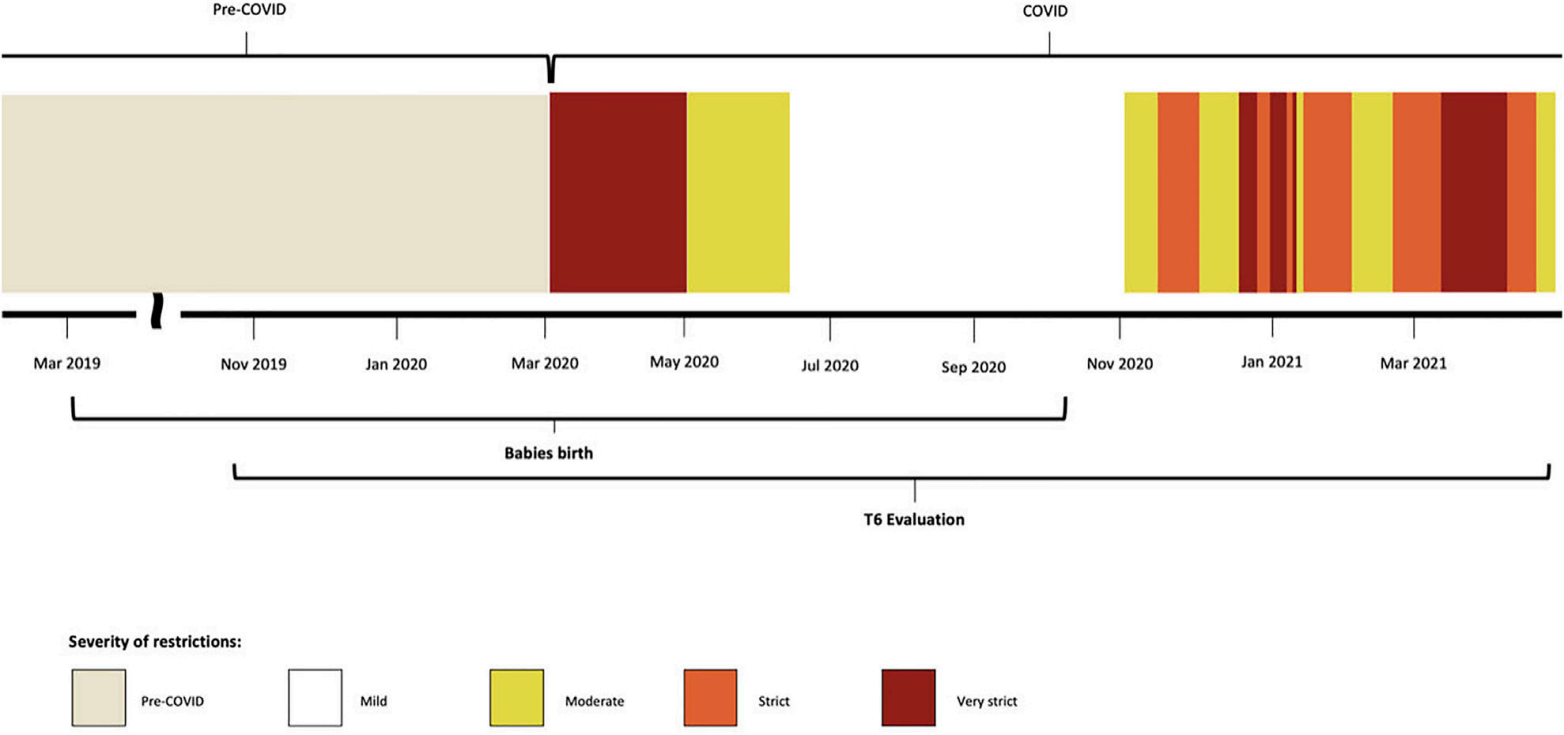
Timeline overview of the study process according to the implementation of different restrictive policies. Different patterns represent the severity of the restrictions put in place in Modena province in the study period. Italy, 2019–2021.

Exposure to the pandemic outbreak was recorded as dichotomous (pre-COVID/COVID) depending on if the evaluation took place before or after 4th March 2020, the date of the first regional “lockdown.” Data on the severity of restriction was registered as categorical data depending on how severe restrictions during the 14 days before the visit were and defined as per local legislation during the observed period. Five levels were defined (none, mild, moderate, strict, very strict) and Figure 1 shows the time period during which they were implement in the study area. Details on each level definition are reported in Supplementary Material.

Considering that 6-month-old newborns are rapidly evolving, we chose to use a 14-day separation window from the evaluation day in order to intercept short-term impact of social restrictions on infants’ global development. However, since we have not found a consensus in literature on how to define the timing of restriction in light of its possible effects on child’s development, we conducted a sensitivity analysis for the severity of restriction changing cut-off classification. To assess consistency, we conducted sensitivity analysis defining the severity of restriction set up on the period of the visit.

#### Covariates

According to the theory of the avenues of learning by Ruth Griffiths [21] and its following revisions [4] child development is a complex process resulting from the interactions of multiple environmental, social and familial factors. Major items interacting in the GSCD paradigm of development are familial involvement in a child’s growth and the social-economical-cultural background of the parents. Moreover, unexpected events, such as social changes and restrictions imposed during the COVID-19 pandemic, can affect children’s abilities to adjust to unknown situations. Accordingly, to adjust for potential confounding effects, we collected data on the educational level, work category, and nationality of both parents which were used as proxy descriptors of respectively cultural, economic, and social background of the family. Data on socio-demographic characteristics of caregivers were collected during the enrollment visit and familial involvement was calculated from “Gender stereotypes, educational relations, and infancies” [22] promoted by the Emilia-Romagna region administered to the caregiver of the infant contextually GSCD evaluation. The “Gender stereotypes, educational relations, and infancies” survey for parents is composed of 35 items focusing on parenting themes from a gender equality perspective.

To specifically examine the relationship between the parents’ cultural background and the baby’s cognitive performance according to the GSCD scales, we defined three educational levels (1 = none/primary/middle school; 2 = high school degree; 3 = University or more).

Working groups have been defined according to European Socio-economic Groups (ESeG) [23] classification composed of 9 categories (managers, professionals, technicians and associated professionals employees, small entrepreneurs, clerks and skilled service employees, skilled industrial employees, lower status employees, retired persons, other not employed persons).

Parental involvement levels have been measured as an ordinal variable ranging from 0 to 3 (0 = insufficient, 1 = minimum, 2 = good, 3 = excellent) according to McBride and Mills model [24] and extracting self-reported data from the 15th item (“How would you describe you and your partner in the relationship with your children?”) of the “Gender stereotypes, educational relations, and infancies” survey.

For more information on covariates level definition and administered questionnaires refer to Supplementary Material.

### Statistical Methods

Unless otherwise stated, categorical variables were summarized by absolute and relative frequencies. Median and interquartile ranges (IQR) were used to show continuous variables in the paper. Full descriptive statistical analysis including mean, median, minimum–maximum, standard deviation, and IQR for continuous variables may be found in Supplementary Material.

We calculated descriptive statistics and frequency tables to highlight the characteristics of the population under study and we stratified our sample into two groups: pre-COVID and COVID. We applied the χ^2^ test to measure differences in the distribution of categorical variables in the two groups. The Mann-Whitney U test was used for the comparison of GSCD subscales and GDS scores between Pre-COVID and COVID groups, as data were not normally distributed as assessed by the Shapiro-Wilk normality test.

To assess the GDS time trend locally weighted regression (loess) was used [25]. Linear regression was used to test if the type of restrictions significantly predicted GDS. Type of restriction was entered in the model as an ordinal 5-level variable starting from the least level of restriction to the highest level. By the theory of the avenues of learning by Ruth Griffiths [21] and its following revisions [26] to control for potential confounders the regression model was adjusted for age, nationality, educational level, working status of both parents, and familial involvement level. Age and sex of infants were not included in the model as all GSCD scores are expressed as DQ standardized scores.

### Missing data Analysis

Missing data patterns were analyzed and, when possible, a complete-case analysis was performed. Less than 3% of the study participants had missing outcome data (3 for GDS given by 2 missing data in subscale D and E, and 1 in subscale C) all in online visits, while all had data for the exposure of interest (time of visit and severity of restrictions). Given literature considerations on the small percentage of missing data [27, 28], bivariate analysis between exposures and outcomes was performed with complete case analysis.

Regarding covariate, data were complete for mothers and fathers’ nationality. Data on age category, education, and work were missing for, respectively, 30, 22, and 21 (29%, 21%, and 20%) fathers. Data on familial involvement were missing for 24 subjects (23%). The missing data pattern suggests that observations were missing at random (MAR). A complete description of missing data analysis may be found in the Supplementary Material.

### Subgroup Analysis and Sensitivity Analyses

#### Subgroup Analysis

Due to pandemic restrictions, some of the visits (n:35) were performed online. Staff tried to accurately reproduce the procedures and assessments of in-person GSCD visits, yet, given the possible bias in test evaluation during online visits, and given the higher variability of the scores producing some outlier scores, we performed a subgroup analysis on infants visited exclusively in presence. This determined the exclusion of infants evaluated in the most restrictive periods (those classified in strict and very strict severity of restriction levels) but gave us the possibility of exclude from the analyses outliers as well.

#### Sensitivity Analyses

In multivariable analyses, to minimize the potential for bias and loss of information due to missing data, multiple imputation using chained equations (MICE) was performed and linear regression with and without MICE was calculated [29]. Missing data analysis and multiple imputation approach are described in Supplementary Material.

Finally, we conducted a sensitivity analysis for the severity of restriction changing the timing of cut-off classification. We have not found a consensus in literature on how to define the timing of restriction in light of its possible effects on child’s development. Given that infants are rapidly evolving, in the primary analysis, we defined the type of restrictions more represented in the 14 days prior to visit. However, to assess consistency, we conducted sensitivity analysis defining the severity of restriction set up on the period of the visit.

Data were analyzed using the statistical software IBM SPSS Statistics software, version 27 [30].

The present study was reported according to Strengthening the Reporting of Observational Studies in Epidemiology (STROBE) guidelines [31].

## Results

We analyzed data collected from 104 infants and their parents. 34 infants underwent 6-month evaluations before the pandemic started and 70 after. Table 1 shows the demographic characteristics of the study population, visit method, age, nationality and socio-economic status of both parents, and familial involvement in child growth. Most infants were 6 or 7 months old (86.5%), female and male children were equally represented. 100% of the visits were conducted in person before the pandemic outbreak, while in the “COVID” group, 50% of the visits were conducted online through video call assessment. More than half of the mothers were older than 35 years (64.4%), 92.3% were of Italian nationality and 71.2% had a high educational level (university degree or Ph.D.). Most women worked as professionals (30.8%) or as clerks and skilled service employees (26.0%). For the fathers, more than half of them were older than 35 years (63.5%), 96.2% were of Italian nationality and 46.8% held a degree (49.4% had a high school diploma). Most of them worked as clerks and skilled service employees (31.3%) or professionals (25.3%), a lower percentage of men worked as lower status employees (16.9%) or small entrepreneurs/managers (13.2%). Most families showed an excellent (75.0%) or good (24.2%) involvement in their children’s evolution and growth.

**TABLE 1:** Demographic-socio-economic characteristics of infants and caregivers and familial involvement expressed in n (%). Italy, 2019–2021.

	Tot (104)	Pre-COVID (34)	COVID (70)	p-value
**Infant characteristics**				
**Age (Baby)**				
5 months	4 (3.8)	0 (0)	4 (5.7)	*0.078*
6 months	54 (51.9)	22 (64.7)	32 (45.7)	
7 months	36 (34.6)	12 (35.3)	24 (34.3)	
8 months	8 (7.7)	0 (0)	8 (11.4)	
9 months	2 (1.9)	0 (0)	2 (2.9)	
**Gender (baby)**				
M	61 (58.7)	22 (64.7)	39 (55.7)	*0.405*
F	43 (41.3)	12 (35.3)	31 (44.3)	
**Visit method**				
In person	69 (66.3)	34 (100)	35 (50.0)	<0.001
Online	35 (33.7)	0 (0)	35 (50.0)	
**Griffiths III Scales of Child Development–Completed scales** ^a^				
Scale A–Foundations of Learning	104 (100)	34 (100)	70 (100)	*0.549*
Scale B–Language and Communication	104 (100)	34 (100)	70 (100)	
Scale C–Eye and Hand Coordination	103 (99.0)	34 (100)	69 (98.6)	
Scale D–Personal-Social-Emotional	102 (98.1)	34 (100)	68 (97.1)	
Scale E–Gross Motor	102 (98.1)	34 (100)	68 (97.1)	
General Development Score (GDS)	101 (97.2)	34 (100)	67 (95.7)	
**Parents characteristics**				
Maternal age (at T6 evaluation)				
≤35 years	67 (64.4)	21 (61.8)	46 (65.7)	*0.827*
>35 years	37 (35.6)	13 (38.2)	24 (34.3)	
**Nationality (mother)**				
Italian	96 (92.3)	31 (91.2)	65 (92.9)	*0.714*
Not Italian	8 (7.7)	3 (8.8)	5 (7.1)	
**Educational level (mother)**				
None to middle school	1 (1.0)	0 (0.0)	1 (1.4)	*0.217*
High school	29 (27.9)	13 (38.2)	16 (22.9)	
University	74 (71.2)	21 (61.8)	53 (75.7)	
**Work category (mother)**				
1. Unemployed	10 (9.6)	6 (17.7)	4 (5.7)	*0.465*
2. Retired persons	0 (0)	0 (0)	0 (0)	
3. Lower status employees	3 (2.9)	1 (2.9)	2 (2.8)	
4. Skilled industrial employees	2 (1.9)	0 (0)	2 (2.8)	
5. Clerks and skilled service employees	27 (26.0)	7 (20.6)	20 (28.6)	
6. Small entrepreneurs	15 (14.4)	6 (17.7)	9 (12.9)	
7. Technicians and associated professionals employees	12 (11.5)	3 (8.8)	9 (12.9)	
8. Professionals	32 (30.8)	8 (23.5)	24 (34.3)	
9. Managers	3 (2.9)	3 (8.8)	0 (0)	
**Paternal age (at T6 evaluation)**				
≤35 years	27 (26.0)	9 (26.5)	18 (25.7)	*0.330*
>35 years	47 (45.2)	22 (64.7)	25 (35.7)	
Missing	30 (28.8)	3 (8.8)	27 (38.6)	
**Nationality (father)**				
Italian	100 (96.2)	32 (94.1)	68 (97.1)	*0.595*
Not italian	4 (3.8)	2 (5.9)	2 (2.9)	
Educational level (father)				
None to middle school	3 (2.9)	1 (2.9)	2 (2.8)	*0.849*
High school	42 (40.4)	18 (52.9)	24 (34.3)	
University	38 (36.5)	14 (41.3)	24 (34.3)	
Missing	21 (20.2)	1 (2.9)	20 (28.6)	
**Work category (father)**				
1. Unemployed	1 (1.0)	0 (0)	1 (1.4)	*0.069*
2. Retired persons	0 (0)	0 (0)	0 (0)	
3. Lower status employees	14 (13.5)	5 (14.7)	9 (12.9)	
4. Skilled industrial employees	7 (20.6)	1 (2.9)	6 (8.6)	
5. Clerks and skilled service employees	26 (25.0)	5 (14.7)	21 (30.0)	
6. Small entrepreneurs	5 (4.8)	3 (8.8)	2 (2.9)	
7. Technicians and associated professionals employees	3 (3.6)	0 (0)	3 (4.3)	
8. Professionals	21 (25.3)	6 (17.7)	15 (21.4)	
9. Managers	6 (7.2)	5 (14.7)	1 (1.4)	
Missing	21 (20.2)	9 (26.5)	12 (17.1)	
**Familial involvement level**				
None	1 (1.0)	0 (0)	1 (1.4)	*0.723*
Minimum	3 (2.9)	1 (2.9)	2 (2.9)	
Good	16 (15.4)	8 (23.5)	8 (11.4)	
Excellent	60 (57.7)	24 (70.6)	36 (51.4)	
Missing	24 (23.1)	1 (2.9)	23 (32.9)	

a Missing data for Griffiths III Scales of Child Development: 3 for General Development Score (GDS) given by 2 missing data in subscale D and E, and 1 in subscale C.

**FIGURE 2:**
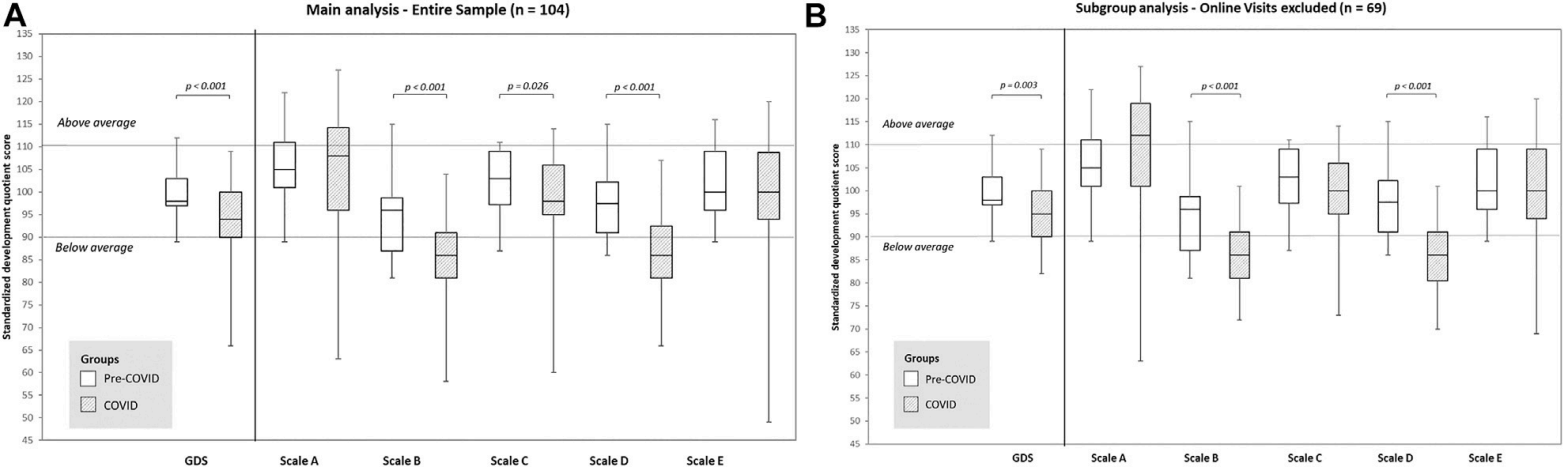
Standardized development quotient scores (DQ) for General Development (GD) and subscales, stratified by evaluation occurring before or after COVID-19 first lockdown. Italy, 2019–2021. Scale A is “Foundations of Learning” scale, Scale B is “Language and Communication” scale, Scale C is “Eye and Hand Coordination” scale, scale D is “Personal-Social-Emotional” scale, scale E is “Gross Motor” scale. Horizontal lines indicate cutoff value of DQ standardized score above and under the average range as calculated for the Italian general population of the same age and sex, according to the GSCD procedure manual [20]. **(A)** Entire sample. **(B)** Subgroup analysis excluding online visits.

As reported in Figure 2A, both global and single scale standardized scores appeared more widespread in the COVID group than in the pre-COVID group. Further, in this group GSCD scores (median: 94; IQR: 90–100) were significantly lower than those observed in the pre-COVID group (median: 98; IQR: 97–103; Mann-Whitney *U* test *p* < 0.001). Differences in score distribution become particularly relevant for subscales B and D (exploring respectively “Language and Communication” and “Personal-social-emotional” areas). Details on subscale score distributions according to period of the assessment are reported in Supplementary Material. As reported in Table 2, a significantly higher number of 6-month infants in the COVID group showed scores below the average, as defined by the normative scoring values for the Italian population [20], both for the global score (22.4% vs. 8.8%, χ^2^ test *p* < 0.05) and for the specific scale scores. The main differences were observed for subscales B (“Language and Communication”) where 70% and 26.5% scores below the average were observed respectively in COVID and pre-COVID group (χ^2^ test *p* < 0.001) and for scale D (“Personal-social-emotional” area) where 60.3% of the COVID group scores lies below the average while only 5.9% of scores are included in the same category for the pre-COVID group (χ^2^ test *p* < 0.001). More details on these findings are reported in Supplementary Material.

**TABLE 2:** Standardized development quotient scores (DQ) per specific subscale and for the global development score (GDS). Italy, 2019–2021.

	DQ Score	TOT n (%)	Pre-COVID n (%)	COVID n (%)	p-value
Scale A	Below Average	10 (9.6)	1 (2.9)	9 (12.9)	0.022
	Average	54 (51.9)	24 (70.6)	30 (42.9)	
	Above average	40 (38.5)	9 (26.5)	31 (44.3)	
	Tot n	104	34	70	
Scale B	Below Average	58 (55.8)	9 (26.5)	49 (70.0)	<0.001
	Average	45 (43.3)	24 (70.6)	21 (30.0)	
	Above average	1 (1)	1 (2.9)	0 (0)	
	Tot n	104	34	70	
Scale C	Below Average	12 (11.7)	3 (8.8)	9 (13.0)	0.644
	Average	82 (79.6)	27 (79.4)	55 (79.7)	
	Above average	9 (8.7)	4 (11.8)	5 (7.3)	
	Tot n	103	34	69	
Scale D	Below Average	43 (42.2)	2 (5.9)	41 (60.3)	<0.001
	Average	57 (55.9)	30 (88.2)	27 (39.7)	
	Above average	2 (2.0)	2 (5.9)	0 (0)	
	Tot n	102	34	68	
Scale E	Below Average	13 (12.7)	2 (5.9)	11 (16.2)	0.178
	Average	79 (77.5)	30 (88.2)	49 (72.1)	
	Above average	10 (9.8)	2 (5.9)	8 (11.8)	
	Tot n	102	34	68	
GDS	Below Average	18 (17.8)	3 (8.8)	15 (22.4)	0.040
	Average	81 (80.2)	29 (85.3)	52 (77.6)	
	Above average	2 (2.0)	2 (5.9)	0 (0)	
	Tot n	101	34	67	

A three classes stratification of the scores is shown: Below average (DQ < 90), Average (DQ = 90–109), Above average (DQ ≥ 110), adapted from the seven groups classification of the Griffiths III manual [4, 15]. Scale A is “Foundations of Learning” scale, Scale B is “Language and Communication” scale, Scale C is “Eye and Hand Coordination” scale, scale D is “Personal-Social-Emotional” scale, scale E is “Gross Motor” scale.

**FIGURE 3:**
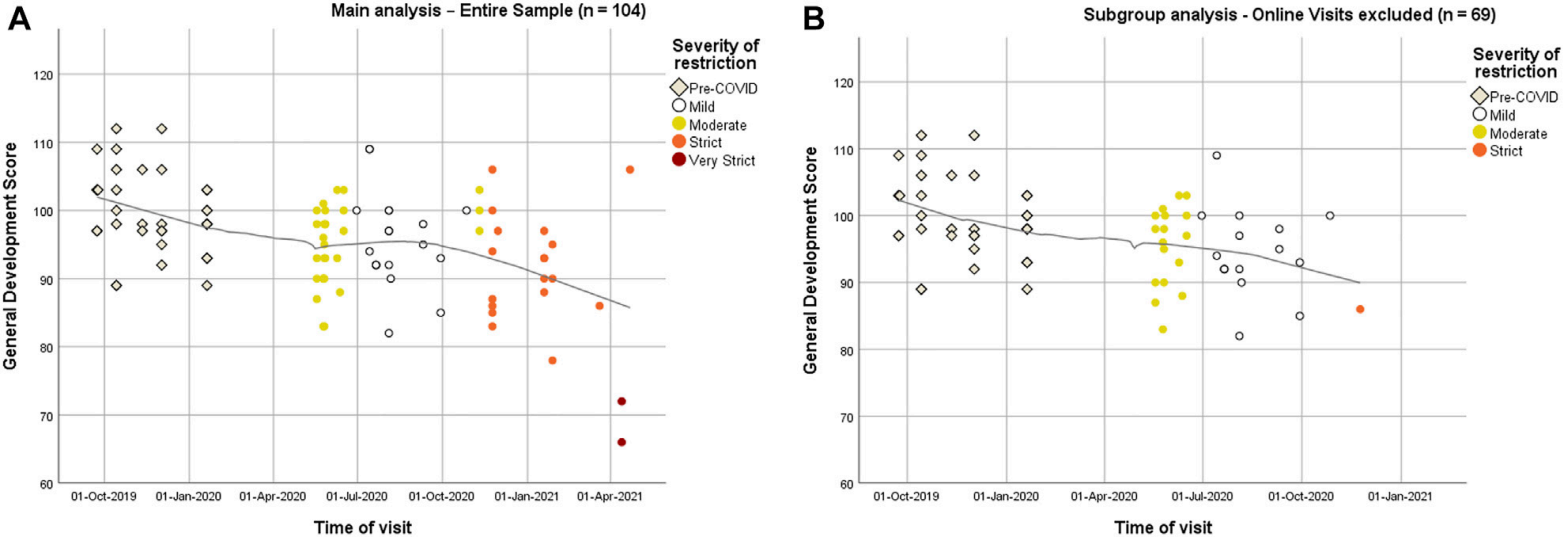
Time trend of standardized development quotient scores (DQ) for General Development (GD). Different coloring represents the severity of restriction occurring 14 days prior to evaluation. Italy, 2019–2021. **(A)** Entire sample. **(B)** Subgroup analysis excluding online visits.

Figure 3A shows the GDS scores time trend. Data show a decreasing trend in GDS in infants evaluated since the start of the pandemic. Locally weighted regression shows a slight increase of scores during the period of least restrictions (White zone) and a rapid decrease with the imposition of more severe local restrictions.

Simple linear regression was used to test if the type of restrictions significantly predicted GDS (Table 3). The overall regression was statistically significant (Adj.R^2^ = 0.237, *p* < 0.001).

**TABLE 3:** Linear regression models testing if the severity of restriction predicts GDS. Italy, 2019–2021.

	Coefficient (β)	95% CI for β	p-value	F-statistic	Adj.R^2
**Main analysis**					
Simple Regression	−3.249	−4.411 to −2.087	<0.001	30.775	0.237
Multiple Regression^a^	−3.699	−5.686 to −1.712	0.003	3.380	0.331
Pooled Multiple Regression with MICE^b^	−3.642	−4.495 to −2.359	<0.001		
**Sensitivity analysis changing timing for type of restriction**					
Simple Regression	−3.181	−4.406 to −1.957	<0.001	26.563	0.204
Multiple Regression^a^	−3.409	−5.596 to −1.222	0.003	2.796	0.272
Pooled Multiple Regression with MICE^b^	−3.394	−5.601 to −2.862	0.004		

Main analysis and Sensitivity analysis after changing timing for cutoff in the severity of restriction classification. Simple regression was conducted with a complete-case analysis. Multiple regression was adjusted for age, nationality, educational level, working status of both parents, and familial involvement level and conducted both with complete case analysis before imputation and then after multiple imputation.

a Multiple regression with complete-case analysis.

b Multiple regression after multiple imputation using chained equations (MICE).

To adjust for potential confounders the regression model was adjusted for age, nationality, educational level, working status of both parents, and familial involvement level. The overall regression was statistically significant (Adj.R^2^ = 0.331, *p* = 0.003). and the worsening of the type of restrictions continued to be associated to a significant decrease in GDS (*p =* 0.001) while other covariates did not significantly predict GDS. Finally given the high percentage of missing data in covariate variables (24% of the sample), multiple regression was performed after multiple imputation and pooled data from linear regression from fifty-five imputed datasets using chained equations (MICE) showed how the severity of restriction continued to significantly affect GDS (*p* < 0.001). Covariates continued to be not significantly associated to GDS.

### Subgroup Analysis and Sensitivity Analysis

Given the possible bias in test evaluation during online visits, we performed a subgroup analysis on infants visited exclusively in presence. As reported in Figure 2B results observed in the whole cohort were confirmed: infants assessed during COVID pandemic showed lower scores than those evaluated in pre-COVID period in all scales and especially in sub-scales B and D, respectively Language and Communication and Personal-Social-Emotional subscale. Details on subscale scores for this subgroup of infants are reported in Supplementary Material. Figure 3B shows a similar over-time decreasing pattern as seen with the entire cohort.

Pooled data from multiple linear regression after MICE showed that the level of restriction continues to significantly affect GDS (*p* = 0.009). Covariates did not significantly predict GDS.

Finally, we conducted a sensitivity analysis for the type of restriction changing the cut-off classification of restriction from defining zones as the most representative restriction during the 14 days prior to visit defining zones based on the restriction rules set up on the period of the visit. Results of regression are similar even if with slightly worse performance and are shown in Table 3.

## Discussion

High media and scientific attention raised on the socio-psychological consequences of the COVID-19 pandemic [32, 33] and the World Health Organization (WHO) has identified mental health as an integral component of the COVID-19 response [34].

This pandemic has undermined the sense of security of the population, putting families in economic difficulty and completely changing their daily lives. During quarantine, many children experienced isolation periods and were not allowed to meet friends, relatives and other people outside the household. Since in infants social and language development is widely influenced from social background, it is important to understand the consequences of such changes on newborns.

Using data collected for an ongoing cohort study, we evaluated the general child development, as assessed by Griffiths Scales of Child Development, in six-month-old healthy infants which were assessed partly before the pandemic and partly during the pandemic restrictive social distancing measures. Our results suggest an association between social distancing measures and reduction in Griffiths Scales of Child Development scores as significantly lower scores both in General Development and in subscales were observed in the children assessed during the pandemic period. Scores below-average as reported by GSCD normative scoring tables for the Italian population [20] were observed mainly in “Language and Communication” and “Personal-social-emotional” areas. Time trend analysis showed a decrease in global score over time that seems to follow the severity of social distancing restriction. This last result was observed also when linear regression analyses were performed.

The results of our study must be considered in light of its limitations. Firstly, given the unexpected nature of the pandemic, subject enrollment and sample size were not designed to answer this specific question. The 6-month infants evaluated in this study, actually, were originally enrolled in a longitudinal birth cohort study aimed at investigating the relationship between environmental exposure to phthalates and anthropometric and neurocognitive development in Italian children during the first 3 years of life. The occurrence of the COVD pandemic during the 6-month follow up of the cohort, gave us the opportunity to describe and compare child global development in six-month-old healthy infants exposed to different levels of social restrictions. As the cohort follow up study is still going on, we will have as well the opportunity to follow the changes over time of this specific score. Secondly, familial involvement levels were not assessed directly: a selection of self-reported information collected in the “Gender stereotypes, educational relations, and infancies” survey was used as proxy measures of parental involvement in infant growth. The McBride and Mills Categories of Parental Involvement scheme [24] is the theoretical model that we used to derive familial involvement covariate. According to Ruth Griffiths’ avenues of learning paradigm [21] family engagement, along with relational coherence, self-care, and attachment style, is a primary component determining the child’s social-emotional development. In our study, we analyzed only the first of these aspects, with no contextual analysis of parenting style or of psychological well-being, stress, or depressive symptoms of parents. This shortcoming could have reduced the validity of our findings. Thirdly, given the confinement measures introduced during the pandemic, we did not perform all the GSCD evaluation through visits in person: the online visits represent an unconventional way to administer the Griffiths III Scales of Child Development and it must be noted. However, sensitivity analysis performed excluding online visits showed consistent results. Finally, a possible selection bias should be recognized since it was not possible to verify whether mothers and families who took part in the study differed in their socioeconomic, familial, and demographic characteristics from those who did not choose to participate. The demographic characteristics of the infants and the parents in our sample are congruent with those of the population in the Emilia-Romagna region as for infant sex, parent’s nationality, and parent’s occupational status [35, 36]. However, data we recorded from both mothers and fathers show that they have a higher educational level than the average in the Emilia-Romagna area making our results more applicable in a context with a higher level of education.

In a recent preprint work, Deoni et al. described initial signs of substantial cognitive and performance deficits in infants born during the pandemic and suggested that early development is impaired by the environmental conditions brought on by the pandemic [37]. Early findings of altered temperament in 3-month-old infants are also provided by Provenzi et al. [26] Our results show that most of the examined infants are in line with the national average rates indicative of regular development. Still, a significant reduction of the GDS was recorded (from a median standardized score of 98 to a median standardized score of 93). Overall, we found that subscales evaluating “Language and Communication” and “Personal-social-emotional” areas seem to be most affected by the pandemic outbreak.

This finding is consistent with the analysis of the major developmental milestones of infants [38]: language and social-emotional abilities flourish up to 2 years of age, with external stimuli serving as the primary drivers of such development. In particular, in the “Prespeech” stage of language development, receptive language is crucial, and sound localization skills are substantially improved. During protracted periods of isolation this set of external stimuli risk to become very poor.

On the other hand, it should be noted that during quarantine, some couples or families may have paid greater attention to their babies, spending more time with them and enriching their home environment with more stimuli and this could have, at least partially, counterbalanced the negative effects on infant general development of the social restrictive measures [39, 40].

As for the social and emotional aspects of neurodevelopment, the situation is even more tangled. During lockdown, parents may have experienced low mood and other psychological symptoms [6], which may have influenced their perception of child development. According to a recent study [41], parents tended to be particularly concerned about their children’s development during lockdown, and a “negative influence” on the children’s social-emotional development coming from the parents themselves cannot be excluded.

Considering that a child’s growth is a continuous process constantly influenced by life experiences and environmental background, our results seem to suggest that infants are not immune to the adverse effects of the enacted restriction on cognitive development and mental health.

Our findings also seem to show that the severity of the restrictions negatively affects the infants’ scores, as shown in the results of linear regression: the more the degree of restriction increases, the more the GDS decreases. It is hard to find studies examining specifically the strength of social isolation measures during COVID-19 as a predictor of adverse development outcomes in healthy infants. Therefore, to the best of our knowledge, our paper is the first to describe a preliminary overview of how GDS scores in 6 months old infants decrease over time in relation to the severity of the social restrictions in place. This result must be considered with all its limitations, since, to date and with our dataset, it is difficult to untangle the potential specific factors involved in the decrease of scores over time observed after the pandemic outbreak. Further, it is difficult to define and identify the most susceptible time window potentially affecting child development. Since we have not found a consensus or specific suggestions in literature and, considering that 6-month-old newborns are rapidly evolving, we chose to use a 14-day separation window from the evaluation day in order to intercept short-term impact of social restrictions on infants’ global development. However, a cumulative effect, related to the length of time the infants spent living in the “pandemic era,” could be hypothesized as well. As widely stated, the relevance of familial and socio relationships as environments for early socio-personality development is crucial [38] and, during the pandemic, confinement measures have led to an important social deprivation. The first 1,000 days of a child’s life represent a highly sensitive moment for child development and growth [42] and our findings highlighted that there are specific areas of a child’s development that are more vulnerable than others when infants are affected by confinement measures.

These findings strengthen the need for additional research specifically designed in order to identify and address the specific risk and protective factors potentially involved in adverse developmental outcomes in early preschoolers exposed to isolation periods.

From a public health point of view, it appears important to fill the knowledge gap surrounding the short and long-term impacts of the current pandemic on infant and children’s general development. Public health decision-makers are called to balance the necessity for SARS-CoV-2 spread control and the effect that said measures have on the population. As outlined by Deoni et al. [37] this lack of information slows the development of evidence-based strategies to follow-up sensitive infants or informed guidance for school and daycare reopening.

## Data Availability

The data underlying this article will be shared at reasonable request to the corresponding author.
