# Supplementary material for: The Kids Are Alright (?). Infants’ Development and COVID-19 Pandemic: A Cross-Sectional Study
Source: Int J Public Health. 2022 Jun 20;67:1604804. doi: 10.3389/ijph.2022.1604804 (PMC9252310; doi:10.3389/ijph.2022.1604804)
Supplement: Supplementary file 1 [file DataSheet1.PDF]

The kids are alright (?). Pandemic social restriction measures and infants' development: a cross-sectional study.

Supplementary Material

I. Restrictions and local laws - the Italian “System of colors”

Categories of social distancing measures in place in Modena district during the period of assessment (from September 23, 2019, to April 22, 2021) are defined as follows:

Pre-COVID Group	None	Before the pandemic outbreak, until March 1st, 2020(1)
COVID Group	Mild	Phase Three(2) or White Zone(3) measures in place
	Moderate	Phase Two(4) or Yellow Zone(5–8) measures in place
	Strict	Orange Zone(9) measures in place
	Very strict	Lockdown(10) or Red Zone(11) measures in place

The main features of the different restrictive measures are listed below:

Mild restrictions include:

Phase 3

- Freedom of movement in and out of regions
- Obligation to wear a mask when on public transport vehicles, and indoors
- Schools are closed; universities use remote learning platforms
- Stop fairs and congresses, stop events and sports competitions
- Gyms are opern
- Shops, coffees, and restaurants are open
- The activities of summer camps for children indoors or outdoors are allowed
- Smart working is not mandatory, but strongly recommended

OR

White zone

- Allowed movements between white or yellow regions without time and motivation limits. If equipped with a green pass it is possible to move anywhere even in the orange or red area.
- Shops, coffees and restaurants are open; maximum 6 people per table indoors.
- All other activities resume, in compliance with some basic rules: mask indoors, interpersonal distancing, hand hygiene.
- Obligation to wear a mask when on public transport vehicles, and indoors

Moderate restrictions include:

Phase two

- The obligation of self-certification remains in force
- It is possible to move to visit the relatives
- Outdoor motor activity and walks in the parks are allowed
- Schools keep being closed
- Commercial activities gradually reopen, last are hairdressers and beauty centers
- Coffees and restaurants reopen
- The activities of the construction and manufacturing sector gradually resume after the lockdown
- Public transport works with reduced seats
- Obligation to wear a mask when on public transport vehicles, and indoors

OR

Yellow zone

- Curfew from 10 pm to 5 am,
- It is possible to leave home only for work or emergencies
- Coffees and restaurants are open from 5 am to 6 pm, takeaway is suspended after 10 pm, home delivery is always possible.
- On weekends shops are closed, except for groceries, pharmacies, newsagents and other essential services.
- Museums and exhibitions are closed
- Remote learning is mandatory in high schools and universities, schools are open for lower grade students.
- Public transport vehicles may be filled to a maximum of 50% of their capacity.
- Gyms, swimming pools, theaters and cinemas are closed
- Obligation to wear a mask when on public transport vehicles, and indoors

Strict restrictions include:

Orange zone

- Curfew from 10 pm to 5 am
- It is possible to leave home only for work or emergencies
- Movement in and out of one region to another and from one municipality to another is prohibited unless there are proven reasons. Avoid unnecessary movement even within your municipality
- Coffees and restaurants are closed, home delivery is always possible.
- On weekends shops are closed, except for groceries, pharmacies, newsagents, and other essential services.
- Museums and exhibitions are closed
- Remote learning is mandatory in high schools and universities, schools are open for lower grade students.
- Public transport vehicles may be filled to a maximum of 50% of their capacity.
- Gyms, swimming pools, theaters and cinemas are closed
- Obligation to wear a mask when on public transport vehicles, and indoors

Very strict restrictions include:

Red zone

- It is forbidden to travel, even within one's own municipality, at any time, unless proven reasons of work, necessity, health; movement from one region to another and from one municipality to another is prohibited.

- Coffees and restaurants are closed, home delivery is always possible.
- All shops are closed, except for groceries and other essential services.
- Museums and exhibitions are closed
- Remote learning is mandatory in high schools and universities, and in middle school 2nd and third class; schools are open for lower grade students.
- Public transport vehicles may be filled to a maximum of 50% of their capacity.
- Gyms, swimming pools, theaters, and cinemas are closed
- It is allowed to perform physical activity outdoors, near home
- Obligation to wear a mask when on public transport vehicles, and indoors

OR

Lockdown

- It is forbidden to travel, even within one's own municipality, at any time, unless proven reasons of work, necessity, health; movement from one region to another and from one municipality to another is prohibited. Self-certification is mandatory anytime leaving home.
- Coffees and restaurants are closed, home delivery is always possible.
- All shops are closed, except for groceries and other essential services.
- Museums and exhibitions are closed
- Schools are closed.
- Public transport vehicles may be filled to a maximum of 50% of their capacity (in specific situations even less)
- Gyms, swimming pools, theaters and cinemas are closed
- It is allowed to perform physical activity outdoors, near home; public parks can be closed by the major
- Smart working is allowed in any circumstances, even in derogation from the current regulation
- Obligation to wear a mask when on public transport vehicles, and indoors

Table I: Distribution of patients stratified by type of restrictions.
COVID Group is divided by type of visit (in presence or online visit). Pre-COVID group visits were all performed in presence.

		**Pre-COVID (n: 34)**	**COVID (n: 70)**	
			**In presence (n:35)**	**Online (n:35)**
**Type of restrictions**	*Pre-COVID*	34 (100)	0	0
	Mild	0	16 (46)	3 (9)
	Moderate	0	18 (51)	11 (31)
	Strict	0	1 (3)	19 (54)
	Very Strict	0	0	2 (6)

II. How familial involvement level was calculated

Parental involvement levels have been measured as an ordinal variable ranging from 0 to 3 (0 = insufficient, 1 = minimum, 2 = good, 3 = excellent).

The theoretical paradigm inspiring our classification is McBride and Mills model(13,14), which defines three main categories of mothers' and fathers' parental involvement with their preschool children:

Table II.a: ***Categories of Parental Involvement***

**Category**	**Definition**	**Example**
1. Interaction	Parent and child interacting one-on-one	
a. Play	Parent and child engaged in a "child-centered activity for pleasure (does not include parent watching child play).	Playing a game, building with blocks, etc.
b. Functional	Parent performing or assisting the child in a care task that the child could not complete alone.	Bathing child, dressing child, mealtime, etc.
c. Parallel	Parent and child involved in an adult-centered activity that does not allow parent to give full attention to child<br>or<br>Parent and child involved in different activities yet are in close proximity and interact periodically.	Parent driving car but interacting periodically with child<br>or<br>Parent washing dishes and child playing with a toy by the sink.
d. Transitional	Parent performs some task which assists child in moving from one activity to another.	Dropping off child at child care center, bedtime routine (brushing teeth drink of water, prayers, etc.)
2. Accessibility	Parent may or may not be directly engaged in interaction with children but is both physically and psychologically available to them (all interactions by definition include accessibility).	Children in one room playing with toys and parents in another; if children need parents they can go to them and parents would be available to them.
3. Responsibility	Parent assumes responsibility for the welfare and care of child (does not necessarily include direct contact with child), does not include financial responsibility.	Making child care arrangements, scheduling visit to pediatrician for booster shots, buying child's clothes, etc

Comparing this model to the self-reported data of the 15th item (“*How would you describe you and your partner in the relationship with your children?”*) of the “Gender stereotypes, educational relations and infancies” survey, we performed the following parallelisms:

Table II.b: Category of Parental Involvement according to Mc Bride & Mills model

**Parents defining themselves as…**	**Category of Parental Involvement according to Mc Bride & Mills model**
Patient	Accessibility
Affectionate	Interactions
Protective	Responsibility

It should be noted that the 15th item of the “Gender stereotypes, educational relations and infancies”(15) survey featured two columns, both of which were filled in by one parent, who also reported (2nd column) the partner's style of interaction with the infant, as shown in the example below:

Table II.c: *How would you describe you and your partner in the relationship with your children:*

Me	***mom**/dad*	***My partner***	*mom/**dad***
Patient		*Patient*	*X*
Affectionate	*X*	*Affectionate*	*X*
Protective	*X*	*Protective*	*X*

Considering this, we numerically determined the level of global familial engagement of the families, as follows:

Table II.d: Level of global familial engagement of the families

0	Insufficient	No key characteristics* are present in the family: neither the mother nor the father has one.
1	Minimum	One key characteristic* is present in the family: the mother or the father has it, or they both do.
2	Good	Two key characteristics* are present in the family: the mother or the father has them, or they both do. (The global familial engagement has been recorded as “2”, even when a parent has one characteristic, and the other has another one).
3	Excellent	Three key characteristics* are present in the family: the mother or the father has them, or they both do. (The global familial engagement has been recorded as “3” even when one parent has 2 characteristics* and the other has a different one, or vice versa).

*Key characteristics are **Accessibility, Interactions, Responsibility**.

III. Missing data analysis and Multiple imputation approach

Missing data analysis was performed.

We had complete data for the exposure of interest (Time of visit and Severity of restrictions).Less than 3% of the study participants had missing outcome data (3 for GDS given by 2 missing data in subscale D and E and 1 in subscale C) all in online visits. Given literature considerations on a small percentage of missing data(18,19), bivariate analysis between exposures and outcomes was performed with complete case analysis.

Regarding covariate, data was complete for mothers and fathers' nationality. Data on age category, education, and work were missing for, respectively, 30, 22, and 21 (29%, 21%, and 20%) fathers. Data on familial involvement were missing for 24 subjects (23%). Due to missing covariates, of 104 infants, 61 (58.7%) had data available for all analyzed variables

When applying the multiple regression model, a complete case analysis would discard 24% of the sample mainly due to missing data of covariates. The missing data patterns do not follow a regular pattern, with many participants missing individual covariates.

Missing data analysis was performed, and although missing data was prevalently present in the COVID group, the missing data pattern suggests that observations were missing at random (MAR) and not MNAR (missing not at random) which may be addressed with multiple imputation.

There is no test that may help differentiate between MAR and MNAR, yet the knowledge of our data helps us diagnose the randomness of our sample. Data was prevalently missing in the COVID group because of the difficulties to collect all information from parents (parents not comfortable to come to a hospital setting to perform follow up visits or sending back none or only partial material) during the period of the pandemic outbreak and not given by the missing data in itself (for example retention of information due to sensitive data). This assumption is strengthened by the fact that distribution of missing variables stays similar between both groups and to the general population and that patients were enrolled as representative of the entire population prior to COVID-19 outbreak.

On the assumption that missing observations were missing at random (MAR), to minimize the potential for bias and loss of information from missing data, fifty-five datasets were imputed(20) using chained equations (MICE). The imputation model consisted of variables included in the analysis model: outcome variable (PGS), exposure variable (severity of restrictions), and covariates (age, nationality, educational level, working status of both parents, and familial involvement level). Auxiliary variables included in the imputation model were the Griffiths raw subscales scores and parent characteristics leading to familial involvement level construction.

IV. Standardized development quotient scores (DQ) for General Development (GD) and subscales, stratified by evaluation occurring before or after COVID-19 first lockdown. Italy, 2019-2021.

	n	Mean	SD	Min	25th percentile	Median	75th percentile	Max	*p-value **
**General Development Score**									
Pre-COVID	34	99,74	± 6,102	89	97	98	103	112	
COVID	67	93,57	± 7,89	66	90	94	100	109	<0,001
**Scale A**									
Pre-COVID	34	105,79	± 6,746	89	101	105	111	122	
COVID	70	104,96	± 14,836	63	96	108	114.25	127	0,538
**Scale B**									
Pre-COVID	34	94,68	± 7,223	81	87	96	98,75	115	
COVID	70	85,51	± 8,516	58	81	86	91	104	<0,001
**Scale C**									
Pre-COVID	34	102,38	± 7,08	87	97,25	103	109	111	
COVID	69	98,14	± 9,915	60	95	98	106	114	0,026
**Scale D**									
Pre-COVID	34	97,94	± 6,985	86	91	97,5	102,25	115	
COVID	68	86,32	± 8,925	66	81	86	92.5	107	<0,001
**Scale E**									
Pre-COVID	34	102,06	± 6,742	89	96	100	109	116	
COVID	68	98,6	± 13,543	49	94	100	108.75	120	0,234

Table IV.a Standardized development quotient scores (DQ) for General Development (GD) and subscales, stratified by evaluation occurring before or after COVID-19 first lockdown. Entire Sample. Italy, 2019-2021.

Scale A is “Foundations of Learning” scale, Scale B is “Language and Communication” scale, Scale C is “Eye and Hand Coordination” scale, scale D is “Personal-Social-Emotional” scale,

*scale E is “Gross Motor” scale. *p-value* was calculated with Mann Whitney U test for independent samples.

	n	Mean	SD	Min	25th percentile	Median	75th percentile	Max	*p-value **
**General Development Score**									
Pre-COVID	34	99,74	6,102	89	97	98	103	112	
COVID	34	94,68	6,202	82	90	95	100	109	0.003
**Scale A**									
Pre-COVID	34	105,79	6,746	89	101	105	111	122	
COVID	35	108,17	13,667	63	101	112	119	127	0.155
**Scale B**									
Pre-COVID	34	94,68	7,223	81	87	96	98,75	115	
COVID	35	86,91	6,409	72	81	86	91	101	<0.001
**Scale C**									
Pre-COVID	34	102,38	7,08	87	97,25	103	109	111	
COVID	34	99,66	8,352	73	95	100	106	114	0.150
**Scale D**									
Pre-COVID	34	97,94	6,985	86	91	97,5	102,25	115	
COVID	34	85,41	7,62	70	80,5	86	91	101	<0.001
**Scale E**									
Pre-COVID	34	102,06	6,742	89	96	100	109	116	
COVID	35	100,37	11,186	69	94	100	109	120	0.373

Table IV.b Standardized development quotient scores (DQ) for General Development (GD) and subscales, stratified by evaluation occurring before or after COVID-19 first lockdown. Post-hoc subgroup analysis excluding online visits. Italy, 2019-2021.
*Scale A is “Foundations of Learning” scale, Scale B is “Language and Communication” scale, Scale C is “Eye and Hand Coordination” scale, scale D is “Personal-Social-Emotional” scale, scale E is “Gross Motor” scale. *p-value* was calculated with Mann Whitney U test for independent samples.

V. Standardized development quotient scores (DQ) per scale, stratified by evaluation occurring before or after COVID-19 first lockdown and with a 7 classes stratification of the scores. Italy, 2019-2021.

	DQ Score	TOT n (%)	Pre-COVID n (%)	COVID n (%)	*P value*
Scale A	Extremely Low	4 (3.8%)	0 (0%)	4 (5.7%)	0.079
	At the limit	0 (0%)	0 (0%)	0 (0%)	
	Below Average	6 (5.8%)	1 (2.9%)	5 (7.1%)	
	Average	54 (51.9%)	24 (70.6%)	30 (42.9%)	
	Above Average	27 (26%)	7 (20.6%)	20 (28.6%)	
	High	13 (12.5%)	2 (5.9%)	11 (15.7%)	
	Extremely high	0 (0%)	0 (0%)	0 (0%)	
	Tot n	104	34	70	

Table V.a
Standardized development quotient scores (DQ) in scale A, stratified by evaluation occurring before or after COVID-19 first lockdown and with a 7 classes stratification of the scores. Italy, 2019-2021.

	DQ Score	TOT n (%)	Pre-COVID n (%)	COVID n (%)	*P value*
Scale B	Extremely Low	4 (3.8%)	0 (0%)	4 (5.7%)	<0.001
	At the limit	8 (7.7%)	0 (0%)	8 (11.4%)	
	Below Average	46 (44.2%)	9 (26.5%)	37 (52.9%)	
	Average	45 (43.3%)	24 (70.6%)	21 (30%)	
	Above Average	1 (1%)	1 (2.9%)	0 (0%)	
	High	0 (0%)	0 (0%)	0 (0%)	
	Extremely high	0 (0%)	0 (0%)	0 (0%)	
	Tot n	104	34	70	

Table V.b
Standardized development quotient scores (DQ) in scale B, stratified by evaluation occurring before or after COVID-19 first lockdown and with a 7 classes stratification of the scores. Italy, 2019-2021.

	DQ Score	TOT<br>n (%)	Pre-COVID<br>n (%)	COVID<br>n (%)	*P value*
**Scale C**	Extremely Low	2 (1.9%)	0 (0%)	2 (2.9%)	0.734
	At the limit	1 (1%)	0 (0%)	1 (1.4%)	
	Below Average	9 (8.7%)	3 (8.8%)	6 (13.0%)	
	Average	82 (79.6%)	27 (79.4%)	55 (79.7%)	
	Above Average	9 (8.7%)	4 (11.8%)	5 (7.2%)	
	High	0 (0%)	0 (0%)	0 (0%)	
	Extremely high	0 (0%)	0 (0%)	0 (0%)	
	Tot n	103	34	69	

Table V.c
Standardized development quotient scores (DQ) in scale C, stratified by evaluation occurring before or after COVID-19 first lockdown and with a 7 classes stratification of the scores. Italy, 2019-2021.

	DQ Score	TOT<br>n (%)	Pre-COVID<br>n (%)	COVID<br>n (%)	*P value*
**Scale D**	Extremely Low	2 (2%)	0 (0%)	2 (2.9%)	<0.001
	At the limit	13 (12.7%)	0 (0%)	13 (19.1%)	
	Below Average	28 (27.5%)	2 (5.9%)	26 (38.2%)	
	Average	57 (55.9%)	30 (88.2%)	27 (39.7%)	
	Above Average	2 (2%)	2 (5.9%)	0 (0%)	
	High	0 (0%)	0 (0%)	0 (0%)	
	Extremely high	0 (0%)	0 (0%)	0 (0%)	
	Tot n	102	34	68	

Table V.d
Standardized development quotient scores (DQ) in scale D, stratified by evaluation occurring before or after COVID-19 first lockdown and with a 7 classes stratification of the scores. Italy, 2019-2021.

	DQ Score	TOT	Pre-COVID	COVID	*P value*

		n (%)	n (%)	n (%)	
Scale E	Extremely Low	4 (3.9%)	0 (0%)	4 (5.9%)	0.445
	At the limit	1 (1%)	0 (0%)	1 (1.5%)	
	Below Average	8 (7.8%)	2 (5.9%)	6 (8.8%)	
	Average	79 (77.5%)	30 (88.2%)	49 (72.1%)	
	Above Average	8 (7.8%)	2 (5.9%)	6 (8.8%)	
	High	1 (1%)	0 (0%)	1 (2.9%)	
	Extremely high	0 (0%)	0 (0%)	0 (0%)	
	Tot n	102	34	68	

Table V.e
Standardized development quotient scores (DQ) in scale E, stratified by evaluation occurring before or after COVID-19 first lockdown and with a 7 classes stratification of the scores. Italy, 2019-2021.

	DQ Score	TOT n (%)	Pre-COVID n (%)	COVID n (%)	*P value*
GDS	Extremely Low	1 (1%)	0 (0%)	1 (1.5%)	0.142
	At the limit	2 (2%)	0 (0%)	2 (3%)	
	Below Average	15 (14.9%)	3 (8.8%)	12 (17.9%)	
	Average	81 (80.2%)	29 (85.3%)	52 (77.6%)	
	Above Average	2 (2%)	2 (5.9%)	0 (0%)	
	High	0 (0%)	0 (0%)	0 (0%)	
	Extremely high	0 (0%)	0 (0%)	0 (0%)	
	Tot n	101	34	67	

Table V.f
Standardized development quotient scores (DQ) in GSD, stratified by evaluation occurring before or after COVID-19 first lockdown and with a 7 classes stratification of the scores. Italy, 2019-2021.

References

1. Decreto del Presidente del Consiglio dei Ministri 1 marzo 2020 [Internet]. Available from: https://www.gazzettaufficiale.it/eli/id/2020/03/01/20A01381/sg
2. Decreto del Presidente del Consiglio dei Ministri 11 giugno 2020 [Internet]. Available from: https://www.gazzettaufficiale.it/eli/id/2020/06/11/20A03194/sg
3. Ordinanza del Ministero della Salute 11 giugno 2021 [Internet]. Available from: https://www.gazzettaufficiale.it/eli/id/2021/06/12/21A03664/SG
4. Decreto del Presiente del Consiglio dei Ministri 26 aprile 2020 [Internet]. Available from: https://www.gazzettaufficiale.it/eli/id/2020/04/27/20A02352/sg
5. Ordinanza del Ministero della Salute 5 dicembre 2020 [Internet]. GU Serie Generale, n. 30 del 05-12-2020. Available from: https://www.trovanorme.salute.gov.it/norme/dettaglioAtto?id=77517
6. Decreto-Legge 5 gennaio 2021 n.1 [Internet]. Available from: https://www.gazzettaufficiale.it/eli/id/2021/01/05/21G00001/sg#:~:text=Dal%207%20al%2015%20gennaio,ovvero%20per%20motivi%20di%20salute.
7. Ordinanza del Ministero della Salute 29 gennaio 2021 [Internet]. Available from: https://www.gazzettaufficiale.it/atto/serie_generale/caricaDettaglioAtto/originario?atto.dataPubblicazioneGazzetta=2021-01-31&atto.codiceRedazionale=21A00536
8. Ordinanza del Ministero della Salute 23 aprile 2021 [Internet]. Available from: https://www.gazzettaufficiale.it/eli/id/2021/04/24/21A02503/sg
9. Decreto del Presidente del Consiglio dei MInistri 3 novembre 2020 - Articolo 2 [Internet]. Available from: https://www.gazzettaufficiale.it/eli/id/2020/11/04/20A06109/sg
10. Decreto del Presidente del Consiglio dei Ministri 11 marzo 2020 [Internet]. Available from: https://www.gazzettaufficiale.it/eli/id/2020/03/11/20A01605/sg
11. Decreto del Presidente del Consiglio dei MInistri 3 novembre 2020 - Articolo 3 [Internet]. Available from: https://www.gazzettaufficiale.it/eli/id/2020/11/04/20A06109/sg
12. Franco A, Holý D, Erhartova J, Gallo F, Scalisi P, Lorè B. Final Report of the ESSnet on the harmonisation and implementation of a European socio-economic classification: European Socio-economic Groups (ESeG) [Internet]. Paris: Insee; 2014 Jul [cited 2022 Jan 19] p. 58. Report No.: DG75-F001. Available from: https://circabc.europa.eu/sd/a/519eafb9-186c-4e2c-a178-902f28501ba4/DSS-2014-Sep-08c%20ESSnet-ESeG%20-%20Final%20Report.pdf
13. Pritchett R, Kemp J, Wilson P, Minnis H, Bryce G, Gillberg C. Quick, simple measures of family relationships for use in clinical practice and research. A systematic review. Fam Pract. 2011 Apr;28(2):172–87.
14. McBride BA, Mills G. A comparison of mother and father involvement with their preschool age children. Early Childhood Research Quarterly. 1993 Jan 1;8(4):457–77.
15. Ricerca “Stereotipi di genere, relazioni educative e infanzie” [Internet]. Pari opportunità. [cited 2022 Jan 19]. Available from: https://parita.regione.emilia-romagna.it/documentazione/documentazione-temi/documentazione-stereotipi-di-genere/ricerca-201cstereotipi-di-genere-relazioni-educative-e-infanzie201d-1
16. Abidin R. PSI-4. Giunti Psychometrics;
17. Lamb ME, Pleck JH, Charnov EL, Levine JA. A biosocial perspective on paternal behavior and involvement. Parenting across the life span: Biosocial dimensions. 1987;111–42.
18. Schafer JL. Multiple imputation: a primer. Stat Methods Med Res. 1999 Mar;8(1):3–15.

19. Bennett DA. How can I deal with missing data in my study? Aust N Z J Public Health. 2001 Oct;25(5):464–9.
20. White IR, Royston P, Wood AM. Multiple imputation using chained equations: Issues and guidance for practice. Stat Med. 2011 Feb 20;30(4):377–99.
